# Congenital segmental dilatation of jejunoileal region in a newborn: Unusual clinical and radiologic presentation

**DOI:** 10.4103/0971-9261.71752

**Published:** 2010

**Authors:** M. M. Harjai, A. Katiyar, V. Negi, D. Yadav, M. Sharma

**Affiliations:** Department of Surgery, Army Hospital Research and Referral, Delhi - 110 010, India; 1 Department of Surgery, Army Hospital Research and Referral, Delhi - 110 010, India; 2 Department of Paediatrics and Neonatology, Army Hospital Research and Referral, Delhi - 110 010, India; 3 Department of Radiodiagnosis, Army Hospital Research and Referral, Delhi - 110 010, India; 4 Department of Paediatric Cardiology, Army Hospital Research and Referral, Delhi - 110 010, India

**Keywords:** Neonatal bowel obstruction, segmental dilatation of the ileum, intestinal anomalies

## Abstract

Segmental dilatation of the ileum is one of the uncommon causes of intestinal obstruction in neonates. We present a case of slow transit of bowel contents leading to suspicion of functional bowel obstruction in a new born, which on exploration turned out to be a case of segmental dilatation of the jejuno-ileal region. The clinical and radiological evaluation was suggestive of hypomotility disorder of gut, resulting in diagnostic dilemma and delayed surgical intervention.

## INTRODUCTION

Neonatal bowel obstruction as a result of segmental dilatation of the gut is a very rare disorder with limited published cases.[1–3] The condition is characterized by a sharply defined and markedly dilated segment of the gut flanked by normal caliber afferent and efferent bowel. We present a case in which the emergency exploration of abdomen only solved the diagnostic dilemma.

## CASE REPORT

**Figure 1:**
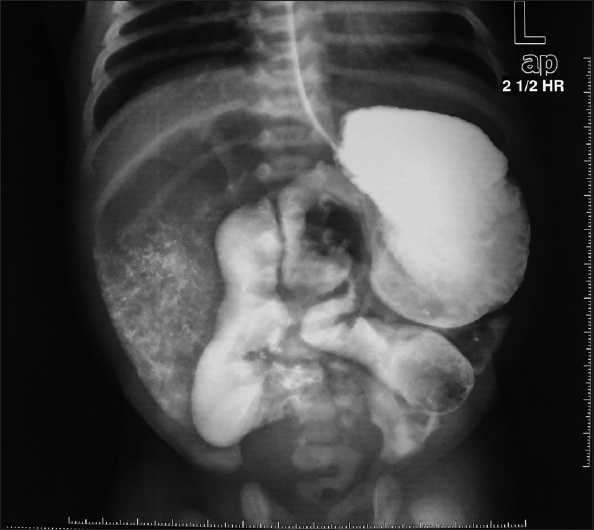
Contrast studies (Barium meal study) showing a dilated gut till the middle part of the small bowel (2½ h film)

**Figure 2:**
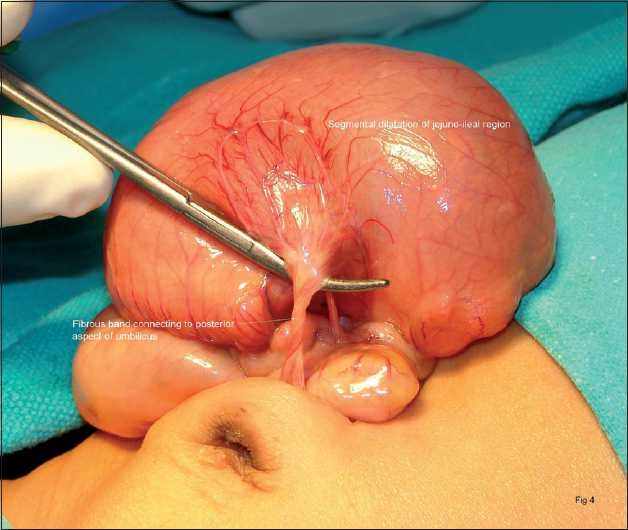
Operative photograph showing sharply demarcated segmental dilatation of the small bowel in line with the lumen about 15 cm in length and the affected section of the intestine dilated regionally to six times the normal value in a divertcular fashion

A routine prenatal sonographic examination in a primigravida of 35 weeks gestation revealed a complex thin-walled cystic mass with internal echogenic debris present in right lower abdomen of the fetus. The cystic mass showed peristalsis and changes appearance during scanning, suggestive of dilated bowel loop. It was opined as a case of anorectal malformation (distal bowel atresia) as peristalsis were appreciated in this segment and it was located posterior to bladder. At birth, the patient passed copious amounts of meconium. He was put on oral feeds but developed bilious vomiting and 120 to 140 ml of light green aspirate every day while on IV fluids. The abdomen has remained soft, there was no distension. Postnatal ultrasound studies did not reveal any intraabdominal cyst. There was no suspicion of malrotation in any of the films or Doppler studies. Barium contrast studies have shown a dilated gut till the mid-portion of the small gut [Figure 1]. The passage of contrast was extremely slow and barium could be seen even in the 60 h film. Though the child was passing stools every other day, he vomited on resumption of feeds. Clinically and radiologically, a hypomotility disorder of gut was suspected. The exploration revealed sharply demarcated segmental dilatation of the small bowel that was in line with the lumen about 15 × 10 cm in size, and the affected section of the intestine was dilated regionally to six times the normal value in a diverticular fashion. The dilated segment was filled with fecal matter in continuity with normal caliber distal bowel and mildly dilated proximal bowel. There was associated malrotation of midgut with cecum lying to the left of umbilicus. A fibrous band was found attached from posterior aspect of umbilicus to the dilated part of the gut [Figure 2]. The treatment consisted of excision of dilated segment, excision of fibrous band and Ladd’s procedure in one stage. Histological examination did not demonstrate any heterotopic gastric mucosa, metaplasia or absence of ganglion cells in the excised specimen.

## DISCUSSION

Congenital segmental dilatation of a portion of the small intestine in neonates causing intestinal obstruction is quite uncommon.[1,2] Segmental dilatation of the ileum is characterized by a sharply defined and markedly dilated segment of the ileum flanked by normal caliber afferent and efferent bowel. Segmental dilatation of the ileum is a rare condition, with only 126 published cases.[3] It can present as an isolated entity as in our case or may be associated with other congenital malformations, gastrointestinal bleeding, anemia, abdominal pain, malabsorption, and growth failure in older children.[4,5] Although it is considered an identical entity involving either colon or small bowel, clinical picture and age of presentation are different. Cases involving the colon have a clinical picture very similar to that of Hirschsprung’s disease, usually appearing in the infancy or later on. Segmental intestinal dilatation is an exceptional pathology with an unknown etiology and a misleading clinical presentation. Several theories were proposed to explain this malformation; however, most authors are rather inclined to an embryological theory incriminating an extrinsic intrauterine intestinal compression.[6,7,8,9,10] In our case we found a band connecting posterior aspect of umbilicus to the dilated part of the small bowel but not found causing compression of gut. Pathological examination shows a bowel wall with all layers as well as a normal ganglion cells. Surgical resection of dilated intestinal segment is curative.

## References

[1] Saha S, Konar H, Chatterjee P, Basu KS, Chatterjee N, Thakur SB (2009). Segmental ileal obstruction in neonates--a rare entity. J Pediatr Surg.

[2] Ojha S, Menon P, Rao KL (2004). Meckel’s diverticulum with segmental dilatation of ileum: Radiographic diagnosis in a neonate. Pediatr Radiol.

[3] Waters KJ, Levine D, Lee EY, Buonomo C, Buchmiller TL (2007). Segmental dilatation of the ileum.Diagnostic clarification by prenatal and postnatal imaging. J Ultrasound Med.

[4] Shah AD, Kovanlikaya A, Beneck D, Spigland N, Brill PW (2009). Segmental dilatation of the ileum in a healthy adolescent. Pediatr Radiol.

[5] Eradi B, Menon P, Rao KL, Thapa BR, Nagi B (2005). Segmental dilatation of ileum: An unusual cause of severe malnutrition. Pediatr Surg Int.

[6] Cheng W, Lui VC, Chen QM, Tam PK (2001). Enteric nervous system, interstitial cells of cajal, and smooth muscle vacuolization in segmental dilatation of jejunum. J Pediatr Surg.

[7] Kobayashi T, Uchida N, Shiojima M, Sasamoto H, Shimura T, Takahasi A (2007). Segmental dilatation of the ileum covered almost entirely by gastric mucosa: Report of a case. Surg Today.

[8] Wurtzel D, Nicosia RF, Yoskovitch A, Zubrow AB (1996). Neonatal intestinal perforation caused by intestinal muscularis defect associated with vascular ectasia. J Matern Fetal Med.

[9] Kella N, Rathi PK (2006). Segmental defect of intestinal musculature: A rare cause of intestinal obstruction in children. J Coll Physicians Surg Pak.

[10] Herlinger H, Maglinte DD (1989). Congenital and developmental anomalies in adolescents and adults.Clinical Radiology of the Small Intestine.

